# *Bacillus subtilis* DSM 32315 Supplementation Attenuates the Effects of *Clostridium perfringens* Challenge on the Growth Performance and Intestinal Microbiota of Broiler Chickens

**DOI:** 10.3390/microorganisms7030071

**Published:** 2019-03-05

**Authors:** Cristiano Bortoluzzi, Bruno Serpa Vieira, Juliano Cesar de Paula Dorigam, Anita Menconi, Adebayo Sokale, Kiran Doranalli, Todd Jay Applegate

**Affiliations:** 1 Department of Poultry Science, University of Georgia, Athens, GA 30602, USA; vieirabs@hotmail.com; 2 Evonik Nutrition & Care GmbH, 63457 Hanau, Germany; juliano.dorigam@evonik.com (J.C.d.P.D.); kiran.doranalli@evonik.com (K.D.); 3 Evonik Corporation, Kennesaw, GA 30144, USA; anita.menconi@evonik.com (A.M.); adebayo.sokale@evonik.com (A.S.)

**Keywords:** *Bacillus subtilis*, broiler, microbiota, necrotic enteritis

## Abstract

The objective of this study was to evaluate the effects of the dietary supplementation of *Bacillus subtilis* DSM 32315 (probiotic) on the performance and intestinal microbiota of broiler chickens infected with *Clostridium perfringens* (CP). One-day-old broiler chickens were assigned to 3 treatments with 8 replicate pens (50 birds/pen). The treatments were: non-infected control; infected control; and infected supplemented with probiotic (1 × 10^6^ CFU/g of feed). On day of hatch, all birds were sprayed with a coccidia vaccine based on the manufacturer recommended dosage. On d 18–20 the infected birds were inoculated with CP via feed. Necrotic enteritis (NE) lesion score was performed on d 21. Digestive tract of 2 birds/pen was collected on d 21 to analyze the ileal and cecal microbiota by 16S rRNA sequencing. Performance was evaluated on d 28 and 42. On d 21, probiotic supplementation reduced (*p* < 0.001) the severity of NE related lesion versus infected control birds. On d 28, feed efficiency was improved (*p* < 0.001) in birds supplemented with probiotic versus infected control birds. On d 42, body weight gain (BW gain) and feed conversion ratio (FCR) were improved (*p* < 0.001) in probiotic supplemented birds versus infected control birds. The diversity, composition and predictive function of the intestinal microbial digesta changed with the infection but the supplementation of probiotic reduced these variations. Therefore, dietary supplementation of *Bacillus subtilis* DSM 32315 was beneficial in attenuating the negative effects of CP challenge on the performance and intestinal microbiota of broilers chickens.

## 1. Introduction

*Clostridium perfringens* (CP) is a natural inhabitant of the poultry intestinal tract that generally lives in balance with other microbial groups, causing no harm to the host [1]. However, because of the higher growth rate of CP when compared to other intestinal microorganisms [2], any factor that disturbs the intestinal homeostasis, especially those that cause cellular damage and/or stimulate mucus production (e.g., coccidiosis), will favor outgrowth of CP [3,4]. When pathogenic strains of CP are established at significant levels in the intestine, signs of necrosis in the intestinal epithelium, hemorrhage, diarrhea and consequently loss of performance may occur [5,6]. Mortality also increases in more severely affected birds [7]. These symptoms characterize an important reemerging disease of modern poultry production: necrotic enteritis (NE).

Until recently, NE and other intestinal diseases remained controlled in broilers by the use of feed antibiotics as growth promoters (AGP). However, the growing concern of the international community on the use of antibiotics in animal production has led to an increase of the so-called antibiotic-free production. One of the consequences of this is an increase in the prevalence of NE in broilers [8]. NE is estimated to cost the poultry industry about U$ 6 billion a year in production losses and control measures [9]. Therefore, significant efforts have been devoted to better understand the epidemiology of NE in broilers and to develop new measures to control field outbreaks of the disease [10,11]. Probiotics are one of the feed additives under investigation for this purpose [12].

Depending on their microbial composition, probiotics can suppress intestinal colonization by pathogenic bacteria by several means, including competition with pathogens for limited nutrients in the intestinal lumen, the production of antimicrobial compounds that directly inhibit the growth of pathogens, the competition for enterocyte binding sites, the stimulation of the host immune system, modulation of the intestinal microbial community and others [8,13,14,15,16]. However, in order to perform these functions in the intestine, probiotic strains need to resist the harsh feeding processing conditions (e.g., high pelleting temperatures) and the non-receptive gastric environment of the host [17,18]. Because of the capacity of *Bacillus subtilis* to form spores and the ability to inhibit the growth of several intestinal pathogens both in vitro and in vivo, this bacterium has been considered one of the most promising microorganisms to be used against the establishment of NE in broilers [15,19,20].

Several mechanisms of action have been described for *Bacillus subtilis*, including competitive exclusion but also by producing antimicrobial peptides that inhibit the growth of some bacterial pathogens [21]. However, different strains or subspecies of *B. subtilis* produce different sets of peptides [22,23,24], which suggests that different *B. subtilis* strains may interact differently with the intestinal microbiota. Among several commercial strains of *Bacillus* used as alternatives to AGP in broilers [23], *B. subtilis* strain DSM 32315 has shown significant positive results on the performance of broilers under *C. perfringens* challenge mainly by improving FCR, decreasing mortality and reducing the load of CP in the ileal digesta [15]. Additionally, Whelan et al. [15] evaluated the cecal microbiota changes associated with *B. subtilis* strain DSM 32315 and CP inoculation and observed that the probiotic supplementation supported beneficial groups of bacteria in the cecal content and reduced potential pathogenic groups; however, Whelan et al. [15] as well as Ma et al. [16] did not evaluate the changes in the ileal digesta microbiota promoted by *B. subtilis* strain DSM 32315. Therefore, the effects of this strain on the diversity, composition and function performed by the microbiota in different sections of the intestine of broilers are still underexplored.

Therefore, we hypothesized that dietary *B. subtilis* DSM 32315 supplementation would improve the performance and reduce the impact of NE on the intestinal microbiota of broiler chickens. The objective of this study was to evaluate the effects of the dietary supplementation with the probiotic *B. subtilis* DSM 32315 on the performance, intestinal lesion score characteristic of NE and the composition, diversity and predicted function of the ileal and cecal microbiota of broiler chickens under CP challenge as a model to reproduce NE.

## 2. Materials and Methods

### 2.1. Birds, Housing and Treatments

A total of 1200 day-of-hatch Cobb 500 male chicks were obtained from the Cobb Vantress Hatchery, Cleveland, GA. Upon arrival at the Southern Poultry Research Group facilities, all the birds were vaccinated by spray cabinet with a commercially approved coccidial vaccine (Coccivac^®^-B52, Merck Animal Health, Kenilworth, NJ, USA) at the manufacturer recommended dosage. Chicks were then allocated in a solid-sided barn and equally distributed in 24 concrete-floor pens covered with new wood-shaving litter (stocking density of 11 birds/m^2^). The animal care and experimental procedures followed the Guide for the Care and Use of Agricultural Animals in Research and Teaching [25], under the supervision of a licensed poultry veterinarian (Study number 3453 17009, Southern Poultry Research Group Internal Review Committee).

Litter was not replaced or amended during the course of this study. Each pen contained one tube feeder and one bell drinker providing *ad libitum* access to feed and water throughout the trial. Thermostatically controlled gas heaters were the primary heat source for the barn but infrared lamps (1/pen) provided supplemental heat during brooding. The lighting program followed the primary breeder recommendations.

Each pen was randomly assigned to one of three treatments, comprising a completely randomized design with three treatments and eight replicates of 50 birds per replicate pen. Treatments groups were: uninfected and non-supplemented group (uninfected); infected and non-supplemented group (infected); infected + supplementation with *Bacillus subtilis* DSM 32315 at 1 × 10^6^ CFU/g of feed (infected + probiotic).

Feed consisted of a non-medicated commercial-type broiler starter (d 0 to 14), grower (d 15 to 28) and finisher (d 29 to 42) diets (Table 1), compounded according to Evonik nutritional recommendations [26]. Diets were fed as crumbles (starter feed) or pellets (grower and finisher feed). In order to represent the most common commercial use of the additive, the sporulated probiotic *Bacillus subtilis* DSM 32315 was included into the feed before pelleting (80 °C; California Pellet Mill, Crawfordsville, IN, USA).

### 2.2. Necrotic Enteritis Experimental Model

The NE experimental model consisted of a coccidial vaccine (Coccivac^®^-B52) at d 0 followed by daily CP (NetB positive strain isolated from a field outbreak) inoculations between d 18–20. In the morning, before the administration of CP, all the birds had their feed and water withdrawn for approximately three hours. After that period, birds on treatments infected and infected + probiotic received a CP inoculum mixed with a measured amount of feed estimated to be consumed within 30 minutes. Birds on the uninfected treatment received a similar amount of their regular feed. Once the feed with CP inoculum was consumed, regular feed and water were restored to birds. *Clostridium perfringens* was added to the feed at a final concentration of 1 × 10^8^ CFU/g.

### 2.3. Data Collection, Sampling Procedures and Laboratory Assays

Birds and feed were weighed weekly by pen to evaluate body weight gain (BW gain), feed intake (FI) and feed conversion ratio (FCR). Mortality was recorded daily and used to correct FCR. On days 21 and 28, three birds per pen were randomly selected, euthanized by cervical dislocation and had their small intestine visually scored for NE lesions. Lesion scoring was based on a 0 to 3 scale [27], as follows: score 0—normal; score 1—slight layer of mucus covering the intestinal mucosa; score 2—necrotic lesions in the intestinal mucosa; score 3—sloughed tissue and blood in the intestinal mucosa or its content.

On d 21, two additional birds per pen were randomly selected, euthanized by cervical dislocation and had their entire gastrointestinal tract (GIT) aseptically collected and stored in ice. Once in the lab, ileal digesta and cecal content were separated, pooled by pen and frozen at −80 °C for further microbiota analysis through 16S rRNA sequencing.

### 2.4. Intestinal Content Total DNA

The ileal digesta and cecal content were subjected to total DNA extraction and purification using a commercial kit according to the manufacturer recommendations (PowerViral Environmental RNA/DNA Isolation Kit–Mo Bio; QIAGEN, Carlsbad, CA, USA), as described by Bortoluzzi et al. [28]. Total DNA quantitative and qualitative analyses were realized by spectrophotometry (NanoDrop 1000 Spectrophotometer; Thermo Fischer Scientific, Wilminton, DE, USA) and agarose gel electrophoresis (1.5%), respectively.

### 2.5. PCR Amplification and Sequencing

The V3-V4 hypervariable region of the 16S rRNA gene was amplified using the primer FwOvAd_341f and ReOvAd_785r, following the procedures previously described by Klindworth et al. [29] and further adapted by Bortoluzzi et al. [28]. The PCR products were cleaned using AMPure XP magnetic beads (Labplan, Dublin, Ireland) and subjected to another PCR to incorporate indexes (Illumina Nextera XT indexing primers, Illumina, San Diego, CA, USA) to the samples. After another cleaning, PCR products were pooled and sequenced at a read length of 300 nucleotides on a MiSeq platform (Illumina, Inc., San Diego, CA, USA).

### 2.6. Bioinformatics

All sequence data processing was performed using the QIIME v. 1.9.1 software package [30]. Sequences were paired-end and quality trimmed using Geneious (Newark, NJ, USA). High-quality sequences were aligned against the SILVA database (Ribocon GmbH, Bremen, Germany), release 119 [31]. The UCHIME software v. 3.0.617, USEARCH v4.2.52 (Tiburon, CA, USA) was used to identify and remove chimeric sequences [32]. Number of sequences per sample was normalized based on the sample with the lowest number of reads for statistical comparison [33]. Operational taxonomic units (OTUs) were assigned at a 97% identity using the SILVA database.

Chao index (minimal number of OTU present in a sample), observed species (OS, number of species present in a community), phylogenetic diversity of the whole tree (PD, minimum length of all the phylogenetic branches required to span a given set of taxa in the phylogenetic tree) and Shannon index (abundance and evenness of the species present in a sample) were calculated as measurements of the intestinal microbiota diversity (alpha diversity). Beta diversity (difference of the intestinal microbiota diversity between treatments) was also accessed. Principal Coordinates Analysis (PCoA) was used to visualize the adjusted data (weighted UniFrac procedure).

To understand the potential implications of NE and probiotic supplementation on the predictive functional profile of the ileal and cecal microbiota, a Phylogenetic Investigation of Communities by Reconstruction of Unobserved States (PICRUSt) was carried out using the Kyoto Encyclopedia of Genes and Genomes (KEGG; Uji, Kyoto, Japan) and the Clusters of Orthologous Groups of proteins (COGs) databases. The PICRUSt analysis is a computational tool that allows the prediction of the functional composition of the bacterial genome based on the 16S rRNA data [34]. For this, a closed-reference OTU table was normalized by the 16S rDNA copy number and the metagenome predicted functions were categorized based on KEGG pathways [35]. The obtained biome file was processed by STAMP (Halifax, Nova Scotia, Canada) version 2.1.3 [36]. For the purpose of this study, only those functions that were significantly different between treatments were presented.

### 2.7. Statistical Analysis

Performance data were analyzed by one-way ANOVA and, in case of significant difference, treatment means were separated by Tukey’s test. Intestinal NE lesion scores, mortality and the frequency of the main bacterial groups observed in the intestinal content were submitted to a non-parametric one-way ANOVA (Kruskal-Wallis test) and, in case of significant difference, treatment means were separated by Dunn’s test. Microbiota diversity indexes were analyzed by pair-wise comparisons using the non-parametric *t*-test performed by QIIME. For all analyses, significance was defined at *p* ≤ 0.05.

## 3. Results

### 3.1. Growth Performance and Necrotic Enteritis Lesion

At d 21, uninfected birds showed no characteristic lesions of NE in the intestine (score 0) but infected broilers had their lesion score increased (*p* < 0.001) to 1.20 on a 0 to 3 scale (Table 2). This condition was partially recovered by probiotic supplementation, which significantly improved birds lesion score to 0.50. No significant differences were detected on intestinal NE lesion scores between treatments at d 28.

Necrotic enteritis impaired BW gain (*p* = 0.004) and FCR (*p* < 0.001) of the broilers from d 1 to 28 (Table 2). Probiotic supplementation during this period partially recovered the FCR but had no effect on BW gain. The negative effects of NE on BW gain and FCR were still detected at d 42 (*p* < 0.001). Probiotic supplementation during the whole experimental period (d 1–42) partially recovered both variables. Feed intake was not affected by the infection nor by the supplementation of probiotic in any of the ages evaluated (Table 2). Overall mortality increased in challenged birds (*p* = 0.005) but probiotic supplementation counteracted this effect (Table 2).

### 3.2. Microbial Diversity of Intestinal Microbiota

In the ileal digesta, NE decreased Chao index (*p* = 0.02), OS (*p* = 0.007) and PD (*p* = 0.03). Probiotic supplementation recovered these effects, increasing the alpha diversity indices to values that were not different to those observed in the uninfected group (Table 3). In the cecal content, only the Shannon index was decreased by NE (*p* = 0.009). Once again, probiotic supplementation restored this effect by keeping the Shannon index at a similar level as that of the uninfected birds.

**Figure 1:**
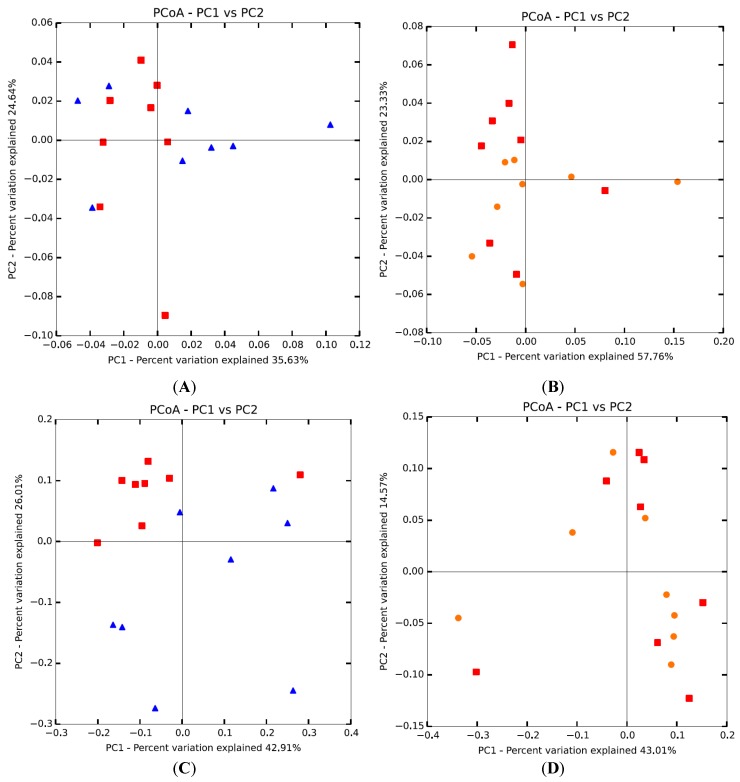
Principal co-ordinates analysis of the diversity of the ileal digesta (**A**,**B**) and cecal content (**C**,**D**) microbiota based on weighted UniFrac distances. Abscissa represents the first principal component, ordinate represents the second principal component. Percentage represents the contribution of the principal component to the total variance. Red square = uninfected group; blue triangle = infected group; orange circle = infected + probiotic group.

When discriminating the microbial communities of the ileal digesta into uninfected and infected birds within the PCoA analysis, the first two coordinates explained 60.27% of the total variance (Figure 1A). By discriminating the microbial communities of the ileal digesta into uninfected and infected + probiotic groups, the first two coordinates explained 81.09% of the total variance (Figure 1B). In the cecal content microbiota, similar comparisons provided explanation for 68.92 and 57.51% of the total variance (Figure 1C,D). Trends of higher separation between groups were visually observed when uninfected and infected birds were compared (Figure 1A,C).

### 3.3. Microbial Composition of Intestinal Microbiota

**Figure 2:**
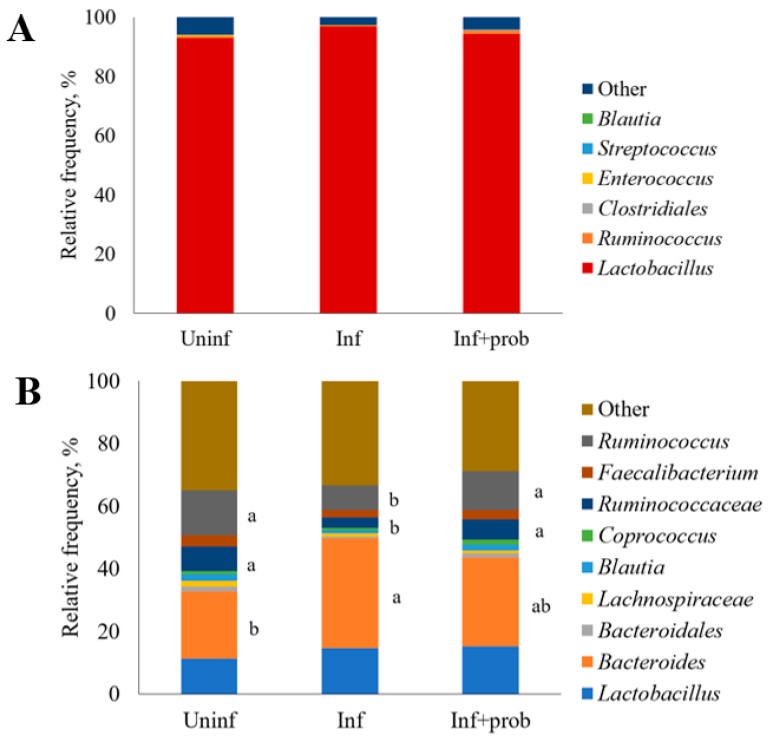
Relative frequency (%) of the main bacterial groups present in the ileal digesta (**A**) and cecal content (**B**) microbiota of broilers challenged with an experimental model to reproduce NE and supplemented with *Bacillus subtilis* DSM 31325. Means of the same bacterial group followed by different letters (a,b) are different by Dunn test (*p* ≤ 0.05).

The ileal digesta microbiota was dominated by bacteria belonging to the phylum *Firmicutes* (99.2%), with a small proportion of *Actinobacteria* (0.5%) and *Bacteroides* (0.1%; data not shown). On a downstream level, the main genera of bacteria observed in the ileal digesta microbiota were *Lactobacillus*, followed by *Ruminococcus*, *Enterococcus*, *Streptococcus* and *Blautia.* The order *Clostridiales* was also representative in the ileal digesta. However, no statistical differences were observed on the relative frequency of these bacterial groups between treatments (Figure 2A).

In the cecal content, the main bacterial groups were *Bacteroides*, *Lactobacillus, Ruminococcus* and members of the family *Ruminococcaceae* but a relevant number of microorganisms could not be properly identified and were grouped in the category “other.” Necrotic enteritis induction decreased the relative frequency of *Ruminococcus* and *Ruminococcaceae* (*p* = 0.01) and increased the relative frequency of *Bacteroides* (*p* = 0.01). Probiotic supplementation, however, increased the frequency of *Ruminococcus* and unclassified members of the family *Ruminococcaceae* and partially restored the frequency of *Bacteroides* to a similar value as that of the uninfected group (Figure 2B).

### 3.4. Predicted Function of Intestinal Microbiota

**Figure 3:**
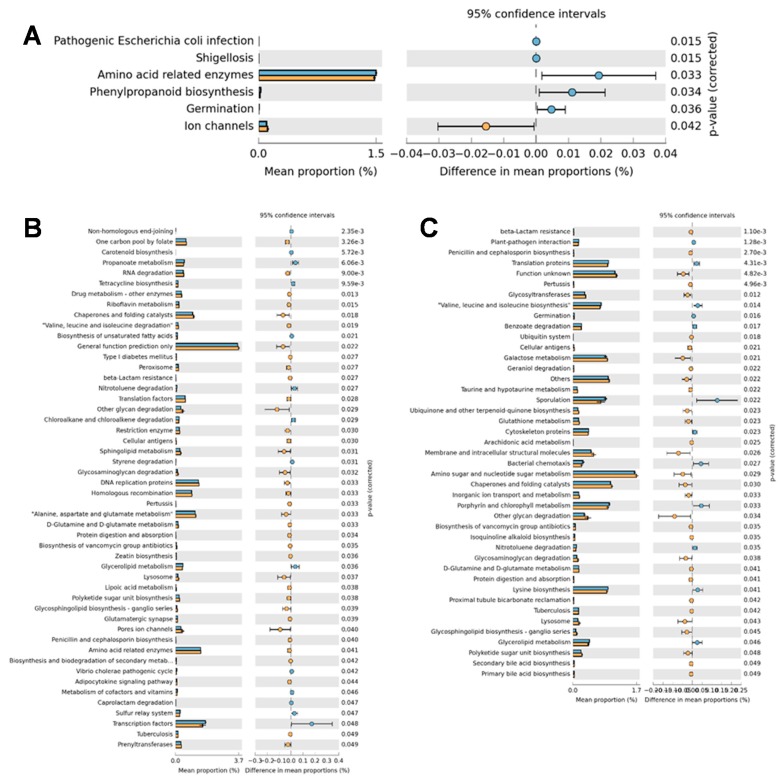
Predicted functions of the ileal digesta (**A**) and cecal content (**B**,**C**) microbiota of broilers challenged with an experimental model to reproduce NE and supplemented with *Bacillus subtilis* DSM 31325. Blue bar = uninfected group; orange bar = infected group (**A**,**B**) or infected + probiotic group (**C**).

As shown by Figure 3A, some changes in the metabolic pathways associated with the ileal digesta microbiota were observed when comparing uninfected and infected birds. Among the functions with larger mean proportions (higher representativeness of genes) within the analyzed metagenomes, those associated with amino acid related enzymes were enriched in the uninfected birds, while “ion channels” category was enriched in the infected group. Because no significant differences were detected in the predicted functional activities of the ileal digesta microbiota between uninfected and infected + probiotic birds (*p* > 0.05), no results were shown for this comparison.

A total of 50 metabolic pathways were significantly different between uninfected and infected birds within the cecal content microbiota (Figure 3B). Among the functions with larger mean proportions, those related to “translation factors,” “DNA replication” and “homologous recombination” were enriched in the cecal content microbiota of infected birds. Necrotic enteritis also enriched “alanine, aspartate and glutamate metabolism” pathways and decreased “glycerolipid metabolism” associated functions.

Less metabolic pathways were identified as different between uninfected and infected+ probiotic groups (Figure 3C). In general, functions associated with “galactose metabolism,” “amino sugar and nucleotide sugar metabolism” and “glycan degradation” were enriched in the cecal content microbiota of birds supplemented with probiotic. On the other hand, “lysine, alanine, leucine and isoleucine biosynthesis” associated functions were decreased in the probiotic supplemented birds.

## 4. Discussion

Overall, the results of this study demonstrated that the dietary supplementation with *Bacillus subtilis* DSM 32315 might be a useful strategy to attenuate the negative effects of NE in broilers. Probiotic supplementation not only improved the growth performance of infected birds but also the severity of lesions characteristic of NE. Part of the explanation for these results might be associated with the ability of the probiotic to sustain an intestinal microbial community, during challenge, similar to that observed in the uninfected birds. Broilers challenged by CP and not supplemented with probiotic had great alterations in the alpha and beta diversity indices of the intestinal microbiota and these modifications promoted relevant changes in the predicted metabolic functions of the microbiota. Both in the ileal digesta and in the cecal content, differences in the diversity, composition and predicted metabolic functions between treatments were improved when birds were supplemented with *Bacillus subtilis* DSM 32315.

At d 28, one week after the end of the CP inoculation, infected birds had BW gain reduced by 12% and impairment of FCR by 8.8%. At d 42, those values were 11.2 and 12.5%, respectively. Probiotic supplementation attenuated 69% of the negative effect of CP infection on FCR at d 28 but did not influence the BW gain. At d 42, probiotic supplementation enhanced 40% of the BW gain and improved the FCR of infected broilers by 33%. Also, probiotic supplementation reduced the severity of the NE lesions found in the intestinal mucosa of infected birds at d 21. This latter finding might have helped the birds to maintain more regular rates of mucosal activity such as the integrity of the intestinal barrier against microbial penetration and nutrient digestive and absorptive mechanisms. Similar results were observed by Whelan et al. [15] when supplementing *B. subtilis* DSM 32315 in broiler infected with CP; these authors observed improvement in the adjusted FCR, mortality and the severity of footpad lesions score. Additionally, in the absence of challenge it has been observed that *B. subtilis* DSM 32315 increased BW gain and FI [16].

As shown by the microbiota diversity indices, richness and evenness of the intestinal microbiota were much more affected by CP infection in the ileal digesta than in the cecal content. Indeed, only the Shannon index of the ileal digesta microbiota was not impaired by CP infection, even though a relevant trend for its reduction (*p* = 0.06) was observed. The higher responsiveness of the ileal digesta microbiota to CP infection is not unexpected, since the main site of CP replication is the small intestine [1,5]. Curiously, only the Shannon index was reduced by CP infection in the cecal content, even though relevant trends for reduction (*p* ≤ 0.10) were observed for all the other diversity parameters.

The diversity of the intestinal microbiota is affected by several factors such as the age of the bird, location in the intestine, diet composition, host’s healthy status and others [37]. It is now speculated that this multiplicity in the microbial composition plays an important role in both heath and disease status of the host [38,39]. The fact that *Bacillus subtilis* DSM 32315 supplementation was efficient in sustaining the alpha diversity indexes of infected broilers at similar levels as those observed in uninfected birds is of great interest and further buttress reasons for the attenuation of the negative effects of NE on broilers performance.

Treating non-infected broilers with the same *Bacillus subtilis* DSM 32315 evaluated here, Ma et al. [16] observed no differences on the diversity of the cecal content microbiota between treated and non-treated groups. However, *Bacillus subtilis* DSM 32315 administration changed the relative frequencies of the main microbial groups present in the cecal content of chickens. Here, we noticed more relevant effects of *Bacillus subtilis* DSM 32315 on the diversity of the microbiota in both ileal digesta and cecal content. Therefore, it seems reasonable to presume that not only the site of the intestine evaluated but also the health status of the broiler, influences the responsiveness of the intestinal microbiota to a probiotic strain.

A great part of the variance in the intestinal microbiota community of broilers (between 57.51 and 81.09%) could be explained by the discriminant analysis. The visual interpretation of the graphics allowed the observation that the separation between treatments was more evident when comparing data from uninfected and infected birds (Figure 1A,C) than it was when comparing data from infected and infected + probiotic birds (Figure 1B,D). This is in agreement with the results of microbial diversity and, indeed, it is a good indicator that the differences between the intestinal microbiota of infected and uninfected broilers were reduced by *Bacillus subtilis* DSM 32315 supplementation. These results gained even more relevance by remembering that data were previously adjusted for PCoA analysis by the weighted UniFrac procedure. The weighted UniFrac is a statistical method that tests the hypothesis that the communities are the same by measuring the distance between them as the fraction of length of the phylogenetic tree branches that lead to descendants from each single community, using the abundance of each microbial group into consideration [40]. Therefore, the chances of mistaken interpretations based on the natural variability of intestinal microbiota were reduced.

As expected, *Lactobacillus* was the genera with greater representation in the ileal digesta microbiota [41]. On average, *Lactobacillus* represented over 93% of the microbial community in this intestinal segment. Treatments did not significantly change the relative abundance of *Lactobacillus* or the other microbial groups that comprise the ileal digesta microbiota. In the cecal content, microbial groups that compose the microbiota were more equally distributed. Within the identified groups, *Bacteroides* was the one with greater relative abundance, followed by *Lactobacillus* and *Ruminococcus*. In contrast to the results of the ileal digesta microbiota, treatments changed the relative abundance of some microbial groups in the cecal content. *Clostridium perfringens* infection increased the relative abundance of *Bacteroides* (21% in uninfected versus 35% in infected birds) and reduced the relative abundance of *Ruminococcus* (14.3% in uninfected versus 7.7% in infected birds) and *Ruminococcaceae* (7.9% in uninfected versus 3.5% in infected chickens). *Bacillus subtilis* DSM 32315 supplementation partially increased the relative abundance of *Bacteroides* (28.3%) to a similar value as that observed in uninfected birds and totally restored the frequency of *Ruminococcus* (12.4%) and members of the family *Ruminococcaceae* (6.4%). On the contrary, Whelan et al. [15] observed that *B. subtilis* DSM 32315 supplementation reduced the relative abundance of the family *Ruminococcaceae* in birds under coccidia plus CP infection but increased members of the family *Lactobacillaceae*, in comparison to the infected and non-supplemented birds. Ma et al. [16] also reported that the treatment with *B. subtilis* DSM 32315 increased the abundance of members of the family *Ruminococcaceae* in the cecal microbiota. Members of the family *Ruminococcaceae*, such as *Faecalibacterium prausnitzii*, have been associated with maintenance of intestinal health mainly by their capacity in degrading complex pant materials [42] and may partially explain the beneficial effects of this probiotic on the performance of the birds. 

Only a few predicted functions of the ileal digesta microbiota were significantly affected by CP infection. The most relevant of them were associated with amino acid metabolism, which were reduced in the infected group. This situation might be associated with the intense protein degradation exhibited by CP strains, which are devoid of metabolic pathways required for the biosynthesis of several amino acids and therefore must be supplied by the environment [3,11]. Moreover, no differences in the predictive functions of the microbiota were detected between uninfected and infected + probiotic groups in the ileal digesta, which corroborates with our previous results showing that probiotic supplementation attenuated the negative effects of CP infection on the diversity and composition of the ileal digesta microbiota and consequently on the growth performance of the broilers. 

On the other hand, several predicted functions of the cecal content microbiota were affected by the treatments. In general, pathways associated with genetic information processing and microbial replication (translation factors, DNA replication proteins and homologous recombination) and protein metabolism (valine, leucine and isoleucine degradation; alanine, aspartate and glutamate metabolism; amino acid related enzymes) were enriched by CP infection, while pathways associated with lipid metabolism (glycerolipid metabolism) were reduced. Probiotic supplementation, on the contrary, enriched pathways associated with carbohydrate metabolism (galactose metabolism; amino sugar and nucleotide sugar metabolism; glycan degradation) and decreased pathways associated with protein metabolism (valine, leucine and isoleucine biosynthesis; lysine biosynthesis). These last results close the set of positive outcomes observed here and associated with the supplementation of *Bacillus subtilis* DSM 32315 to CP infected broilers, in the sense that the probiotic helped to sustain a more desirable microbial metabolism in the ceca, reducing metabolic functions associated with protein fermentation that leads to the production of putrefactive products such as phenols, ammonia and amines, generally considered detrimental to birds performance and intestinal health [43].

## 5. Conclusions

Induction of necrotic enteritis impaired the performance of the broiler chickens and disturbed the intestinal microbiota, by changing the microbial diversity, composition and the predicted functions of their ileal and cecal microbiota. Dietary supplementation of infected broilers with *Bacillus subtilis* DSM 32315 partially restored the performance detriments associated with NE, in comparison to its unsupplemented control counterpart and sustained an intestinal microbiota with diversity and composition more similar to those of uninfected individuals. *Bacillus subtilis* DSM 32315 supplementation also supported the normalization of the predicted functions in the cecal content microbiota of infected broilers, reducing the number of pathways significantly different from those of uninfected birds and assuring a less protein-fermentative profile to the cecal environment.

## Figures and Tables

**Table 1:** Experimental diets composition (as fed basis).

Ingredients (%)	Starter (d 1–14)	Grower (d 15–28)	Finisher (d 29–42)
Corn	50.62	58.78	60.85
Soybean meal, 48% CP	32.30	23.86	20.99
DDGS	8.00	8.00	8.00
Meat and Bone meal	4.00	4.00	4.00
Soybean oil	2.67	2.87	3.97
Dicalcium phosphate	1.12	1.14	1.16
Limestone (CaCO_3_)	0.35	0.40	0.15
NaCl	0.252	0.257	0.258
dl-Met	0.278	0.219	0.196
l-Lys HCl	0.197	0.202	0.177
l-Thr	0.054	0.047	0.037
Choline chrolide, 60%	0.100	0.100	0.100
Vitamin premix ^1^	0.050	0.050	0.050
Trace mineral premix ^2^	0.075	0.075	0.075
Nutrients (%, unless otherwise indicated)			
AMEn (kcal/kg)	2950	3050	3150
Crude protein	23.85	20.44	19.19
SID Lys	1.22	1.02	0.93
SID Met+Cys	0.89	0.76	0.71
SID Thr	0.78	0.66	0.61
SID Trp	0.23	0.18	0.17
SID Arg	1.39	1.15	1.06
SID Ile	0.85	0.71	0.66
SID Leu	1.79	1.59	1.52
SID Val	0.95	0.81	0.76

^1^ Vitamin mix provided the following (per kg of diet): thiamin mononitrate, 2.4 mg; nicotinic acid, 44 mg; riboflavin, 4.4 mg; d-Ca pantothenate, 12 mg; vitamin B12 (cobalamin), 12.0 µg; pyridoxine HCL, 4.7 mg; d-biotin, 0.11 mg; folic acid, 5.5 mg; menadione sodium bisulfite complex, 3.34 mg; choline chloride, 220 mg; cholecalciferol, 27.5 μg; *trans*-retinyl acetate, 1892 μg; α tocopheryl acetate, 11 mg; ethoxyquin, 125 mg; and ^2^ Trace mineral mix provided the following (per kg of diet): manganese (MnSO_4_·H_2_O), 60 mg; iron (FeSO_4_·7H_2_O), 30 mg; zinc (ZnO), 50 mg; copper (CuSO_4_ 5H_2_O), 5 mg; iodine (ethylene diamine dihydroiodide), 0.15 mg; selenium (NaSeO_3_), 0.3 mg.

**Table 2:** Growth performance and intestinal necrotic enteritis (NE) lesion score of broilers challenged with an experimental model to reproduce NE and supplemented with *Bacillus subtilis* DSM 32315.

Treatment	NE Lesion Score		BW Gain (g)	FI (g)	FCR (g/g)	BW Gain (g)	FI (g)	FCR (g/g)	Overall Mort, %
	21 d	28 d	1–28 d			1–42 d			
Uninfected	0.00 ^c^	0.30	1107 ^a^	1717	1.552 ^c^	2464 ^a^	4357	1.769 ^c^	8.0 ^b^
Infected	1.20 ^a^	0.30	975 ^b^	1645	1.689 ^a^	2187 ^c^	4351	1.990 ^a^	16.9 ^a^
Infected+Prob	0.50 ^b^	0.30	1032 ^b^	1645	1.594 ^b^	2298 ^b^	4408	1.918 ^b^	11.1 ^b^
SEM	0.30	0.30	0.061	0.096	0.034	0.074	0.180	0.049	4.10
*p* value	<0.001	0.98	0.004	0.26	<0.001	<0.001	0.836	<0.001	0.005

^a–c^ Means with different superscripts in a column differ (*p* ≤ 0.05) by Dunn’s test (NE lesion score) or Tukey’s test (growth performance measures). Values are means ± SEM of 8 pens. NE = necrotic enteritis, BW gain = body weight gain, FI = feed intake, FCR = feed conversion ratio, mort = mortality, Prob = probiotic, SEM = standard error of mean.

**Table 3:** Diversity of the intestinal microbiota of broilers challenged with an experimental model to reproduce NE and supplemented with *Bacillus subtilis* DSM 31325.

Treatment	Ileal Digesta				Cecal Content			
	Chao	OS	PD	Shannon	Chao	OS	PD	Shannon
Uninfected (A)	198.5	156.6	9.60	2.92	380.9	315.4	23.0	5.90
Infected (B)	150.5	118.4	6.40	2.45	328.9	270.5	19.4	5.30
Infected+Prob (C)	215.9	169.5	10.4	2.88	395.2	326.7	22.9	5.80
*p* value								
A versus B	0.02	0.007	0.03	0.06	0.09	0.10	0.07	0.009
A versus C	0.59	0.64	0.75	0.89	0.33	0.46	0.89	0.76

Chao = minimal number of OTU present in the samples, OS (observed species) = number of species present in the samples, PD (phylogenetic diversity) = minimum length of all the phylogenetic branches required to span a given set of taxa in the phylogenetic tree, Shannon = abundance and evenness of the species present in the samples, Prob = probiotic. *p* values lower than 0.05 imply significant differences by non-parametric *t*-test. Values are means of 8 replicates and a pool of 2 birds/replicate.
